# Trends and impact of government prevention policies on pulmonary tuberculosis notification and mortality in Ningbo, China (2004–2021)

**DOI:** 10.3389/ftubr.2025.1615486

**Published:** 2025-06-20

**Authors:** Tianfeng He, Lin Lin, Xujun Qian, Weitao Yao, Guoxing Li, Jing Huang

**Affiliations:** ^1^Department of Infectious Disease Prevention and Control, Ningbo Municipal Center for Disease Control and Prevention, Ningbo, China; ^2^Department of Economics and Management, School of Public Management, East China Normal University, Shanghai, China; ^3^Department of Primary Health Management, The First Affiliated Hospital of Ningbo University, Ningbo, China; ^4^Department of Occupational and Environmental Health Sciences, Peking University School of Public Health, Beijing, China

**Keywords:** notification, mortality, joinpoint regression, interrupted time series, pulmonary tuberculosis (PTB)

## Abstract

**Background:**

Tuberculosis (TB) is a major global health threat, with Ningbo reporting over 3,000 annual cases of pulmonary tuberculosis (PTB). This study analyzes the notification and mortality rates of PTB in Ningbo from 2004 to 2021.

**Methods:**

We calculated age-standardized annual notification and mortality rates for PTB per 100,000 population and employed interrupted time series regression to evaluate the impact of government policies on these rates.

**Results:**

From 2004 to 2021, Ningbo recorded 68,392 PTB cases and 236 deaths. The unstandardized notification and mortality rates were 51.31 and 0.18 per 100,000 population, respectively. Joinpoint trend analysis showed a significant decline in PTB notification from 2007 to 2016 (APC = −7.3%, *P* < 0.05). Although mortality decreased from 2017 to 2021, this reduction was not statistically significant (APC = −20.5%, *P* = 0.106). Interrupted time series analysis indicated that government policies led to an estimated reduction of three cases in age-standardized PTB notification and about 0.025 deaths per 100,000 population.

**Conclusion:**

Between 2004 and 2021, Ningbo saw a notable decrease in both the notification and mortality rates of PTB due to effective control measures. To achieve the End TB Strategy targets for 2035, it is crucial to enhance efforts in diagnosing and treating PTB—especially among males, adolescents, and older adults.

## 1 Background

TB remains a significant cause of global mortality, substantially impacting public health worldwide. Prior to the COVID-19 pandemic, TB held the position of being the foremost cause of mortality resulting from a solitary infectious agent, surpassing HIV/AIDS in its ranking (1). TB is caused by the bacillus *Mycobacterium tuberculosis* (M.tb), which is transmitted through airborne dissemination of bacteria expelled by individuals suffering from active TB, typically via coughing. The disease primarily targets the pulmonary system, causing TB in the lungs; however, it can also manifest other organs. Most cases (~90%) occur in adults, with higher notification among males. M.tb infection affects approximately one-quarter of the global population (1).

Multiple policies have been implemented to control pulmonary tuberculosis (PTB) spread. In 1995, the World Health Organization (WHO) introduced the Directly Observed Therapy, Short Course (DOTS) strategy as a comprehensive approach to combat TB and improve access to quality care globally. This strategy addressed challenges in diagnosis and treatment through five key components: political commitment with increased funding, case detection through quality-assured bacteriology, standardized treatment with direct observation, uninterrupted drug supply, and robust monitoring systems (2).

The WHO's End TB Strategy was adopted by the 67th World Health Assembly 2014 (3). The 2035 targets encompassed approach: achieving a remarkable 95% reduction in mortality rates compared to 2015, attaining an impressive 90% decrease in TB notification rate, and ensuring that no affected families encounter catastrophic financial burdens due to TB (2, 3). The global cumulative reduction in the notification of PTB from 2015 to 2020 amounted to only 11%, falling short of the milestone set by the End TB Strategy for 20% reduction between 2015 and 2020 (1). Although PTB disproportionately affects middle- and low-income countries, economically developed regions are also vulnerable (4). Thus, development of interventions against the TB epidemic is an urgent public health priority globally (5).

According to the estimation by the WHO, China has witnessed a consistent decline in TB notification. In 2020, China's TB notification rate was estimated at 59 per 100,000 population, placing it lower than that of the other 30 high-burden countries (1). However, China still remains one of the countries with the most severe TB epidemics worldwide (6, 7). In 2020, China had an estimated 842,000 TB cases, ranking second among high-burden countries (1). Ningbo, a city in southeastern China, boasts commendable economic development and experiences an intermediate notification rate of PTB within the country (8). With a population exceeding 9.4 million, Ningbo has reported over 3,000 PTB cases annually in recent years.

To date, there is limited data available on the specific notification and mortality rates of PTB in cities or regions. Furthermore, the trends in PTB notification vary across cities, provinces, and countries, and may even be contradictory. For instance, Cui et al. (9) reported an increasing trend in the standardized reported notification rate of PTB in Xi'an City from 2011 to 2020, which contrasts with the trends observed in China and Zhejiang (2, 10, 11). As Ningbo is a well-developed city along the eastern coast of China, characterized by a large population and a high number of migrant individuals, both of which constitute risk factors contributing to the occurrence of TB. The demographic characteristics of Ningbo are representative of the population composition of well-developed city cities in China. Therefore, our study focused on the notification and mortality rates of PTB in Ningbo from 2004 to 2021 to assess the effectiveness of PTB control projects in the city.

This study utilizes the tuberculosis notification rate as the primary indicator, based on two main considerations. Firstly, while it is essential for incidence data to encompass extrapulmonary tuberculosis, such data can only be acquired through specialized surveys that are often challenging to conduct and financially burdensome. Secondly, in China's infectious disease reporting system, only pulmonary tuberculosis is classified as a legally reportable condition; extrapulmonary tuberculosis falls outside the mandatory reporting framework. Consequently, the reporting data for pulmonary tuberculosis offers advantages in terms of systematic collection and continuity, thereby providing a stable and reliable source of information for trend analysis.

## 2 Methods

### 2.1 Study population and data

The study population comprised Ningbo residents diagnosed with PTB and registered in the Tuberculosis Management Information System (TBIMS), an online platform operated by the China Center for Disease Control and Prevention (CDC), from January 1, 2004, to December 31, 2021. Data on PTB cases and population estimates were sourced from the Ningbo Centers for Disease Control and Prevention. Non-residents and duplicate registrations were excluded from the study. Notification and mortality data were stratified by age groups: 0–4, 5–9, 10–14, 15–19, 20–24, 25–29, 30–34, 35–39, 40–44, 45–49, 50–54, 55–69, 70–74, 75–79, 80–84, and 85 years and above. Raw and age-standardized notification and mortality rates were calculated were calculated using R version 3.5.1 (https://www.r-project.org/). Rates are presented per 100,000 population and standardized according to the 1982 Chinese population and the World Segi population standards, respectively.

### 2.2 Statistical analyses

#### 2.2.1 Jointpoint regression

We analyzed notification and mortality trends using the Joinpoint Regression Program (version 4.9.1.0), developed by the National Cancer Institute.^1^ This method enables detailed trend analysis and calculation of annual percent change (APC), precisely identifying trend shift points. The analysis used natural logarithm of rates as the response variable and diagnosis year as the independent variable. APCs for PTB notification and mortality were estimated using a piecewise linear regression model, assuming a constant rate of change within each segment (2, 12). The grid search method (GSM) was employed to determine the number of joinpoints in the datasets, with up to one joinpoint for datasets containing seven to 11 observations, two for datasets with 12–16 observations, and three for datasets with 17 or more observations (12). The optimal model was selected using the Bayesian Information Criterion (BIC). For each segment of the data, we reported the APC estimates along with their 95% confidence intervals. Statistical significance was determined if the confidence interval did not include zero (*P* < 0.05), with a significance level set at 0.05.

#### 2.2.2 Key policy intervention measures and their time points

Based on a review of existing literature and local health policy documents, this study posits that the following policy interventions may significantly influence the trends in PTB notification. (1) In 2005, Ningbo City implemented a direct reporting system for TB; (2) In 2007, Zhejiang Province initiated the Global Fund Tuberculosis Control Project; (3) In 2011, Ningbo City launched a free screening and HIV co-infection management program targeting the floating population; (4) In 2016, the Ministry of Health of China collaborated with the Gates Foundation to promote molecular diagnostic technologies for TB; and (5) In 2019, COVID-19 prevention and control measures were introduced (including restrictions on gatherings and mandatory mask-wearing). Joinpoint regression analysis will be employed to assess whether these time points represent potential turning points in trends related to PTB notification and mortality.

#### 2.2.3 Interrupted time series regression

Interrupted time series (ITS) analysis is a powerful research design utilized to assess the impact of population-level health interventions introduced at a specific point in time. This approach is increasingly being applied to evaluate a diverse range of interventions, spanning from clinical therapies to national public health policies. In an ITS study, a time series of a specific outcome is utilized to establish an underlying trend, which is then “interrupted” by an intervention at a known point in time.

Our study focuses on the pneumonia prevention policy implemented by the Ningbo government in 2011. In response to the spread of TB among the migrant population, Ningbo initiated several measures. Free TB screenings were offered to this group, along with referrals and follow-ups for suspected cases and confirmed patients. Furthermore, routine TB screenings were conducted among employed migrants, with referrals provided as necessary. Additionally, in the same year, Ningbo launched a program aligned with Zhejiang Province's initiative to control co-infections of TB and HIV. This program intensified efforts to identify, treat, and manage patients with co-infections, aiming to curb the further transmission of both TB and HIV/AIDS.

The dependent variables in this study include the notification and mortality of PTB, standardized for the 1982 population. The independent variables consist of a policy dummy variable that takes the value of one starting in 2011 and 0 for other years, a recentered year variable, and their interaction.

## 3 Results

### 3.1 Crude notification rate and mortality rate

The crude rates of notification and mortality for PTB from 2004 to 2021 were 51.31 and 0.18 per 100,000 population, respectively. Table 1 displays the crude notification and mortality rates of PTB across various age groups. Notably, the highest crude notification rate was observed in the age group 20–24 years, with a rate of 92.80 per 100,000. Conversely, the highest crude mortality rate was recorded in individuals aged 85 years or older, at 2.29 per 100,000. The basic characteristics of tuberculosis patients are described in the Appendix.

**Table 1:** The age-specific crude incidence and mortality rates (per 100,000) of pulmonary tuberculosis in Ningbo, 2004–2021.

**Age group**	**CIR**	**CMR**
0–4 years	0.61	0.07
5–9 years	1.06	0.00
10–14 years	3.80	0.00
15–19 years	51.09	0.05
20–24 years	92.80	0.06
25–29 years	88.34	0.06
30–34 years	67.50	0.05
35–39 years	52.72	0.04
40–44 years	45.43	0.11
45–46 years	40.19	0.09
50–54 years	42.36	0.15
55–59 years	43.21	0.16
60–64 years	52.93	0.26
65–69 years	63.06	0.38
70–74 years	85.21	0.73
75–79 years	81.92	1.30
80–84 years	79.90	2.02
85 years and older	71.14	2.29

CIR, crude incidence rate; CMR, crude mortality rate.

### 3.2 Age-standardized notification and mortality rates by year

Table 2 presents the age-standardized notification and mortality rates for PTB. For comparative purposes, the crude rates before age standardization, using both the China and World standard populations, are also reported. The average age-standardized notification rate, standardized to the Chinese population (ASNRC), was 43.33 per 100,000, while the rate standardized to the World population (ASNRW) was 44.25 per 100,000. Similarly, the average age-standardized mortality rate standardized to the Chinese population (ASMRC) was 0.10 per 100,000, and the rate standardized to the World population (ASMRW) was 0.12 per 100,000.

**Table 2:** The age-standard incidence and mortality rates (per 100,000) of pulmonary tuberculosis, 2004–2021.

**Standardized population**	**Ningbo**		**China standard population**		**World standard population**	
**Year**	**CIR**	**CMR**	**ASIRC**	**ASMRC**	**ASIRW**	**ASMRW**
2004	53.66	0.10	44.02	0.07	46.41	0.07
2005	63.75	0.21	53.96	0.11	55.65	0.14
2006	62.38	0.13	54.27	0.08	55.55	0.10
2007	73.34	0.22	63.93	0.12	65.48	0.15
2008	68.88	0.12	60.82	0.13	62.18	0.16
2009	65.56	0.21	58.98	0.11	59.71	0.14
2010	60.62	0.28	56.68	0.17	56.92	0.22
2011	55.48	0.21	49.33	0.12	49.39	0.17
2012	56.08	0.16	45.03	0.10	45.67	0.12
2013	49.29	0.10	39.82	0.07	40.42	0.08
2014	49.06	0.17	38.92	0.11	39.72	0.13
2015	45.32	0.20	35.60	0.11	36.75	0.13
2016	41.85	0.23	33.15	0.11	33.97	0.14
2017	43.26	0.25	33.93	0.12	34.93	0.16
2018	43.17	0.17	33.21	0.08	34.53	0.11
2019	43.77	0.24	37.87	0.09	38.44	0.12
2020	36.41	0.08	32.16	0.04	32.60	0.05
2021	32.80	0.12	27.99	0.05	28.53	0.06
Total	51.31	0.18	43.33	0.10	44.25	0.12

CIR, crude incidence rate; CMR, crude mortality rate; ASIRC, age-standard incidence rate by Chinese Standard Population; ASMRC, age-standard mortality rate by Chinese Standard Population; ASIRW, age-standard incidence rate by World Standard Population; ASMRW, age-standard mortality rate by World Standard Population.

From 2004 to 2021, both the notification and mortality rates exhibited a declining trend after reaching peaks in 2007 and 2010, respectively. Specifically, the age-standardized notification rate (both ASNRC and ASNRW) peaked in 2007 at 63.93 and 65.48 per 100,000, respectively. Similarly, the age-standardized mortality rate (both ASMRC and ASMRW) peaked in 2010 at 0.17 and 0.22 per 100,000, respectively.

### 3.3 The trend of PTB notification and mortality

**Figure 1:**
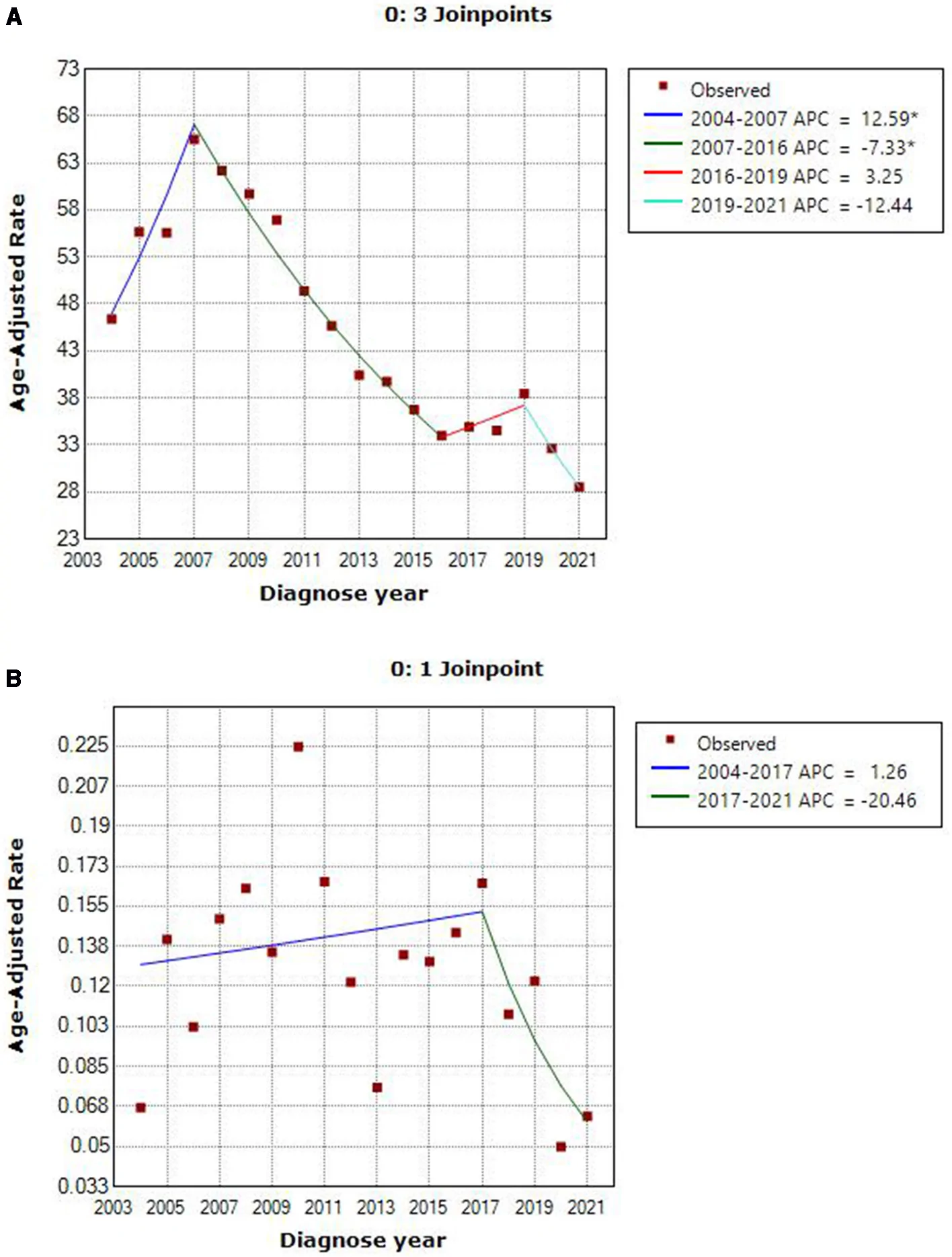
Notification and mortality trend graph of PTB in Ningbo during 2004–2021. *Indicates that the annual percent change (APC) is significantly different from zero at the alpha = 0.05 level. Final selected model: **(A)** 3 joinpoints. **(B)** 1 joinpoint.

Our study examined the trend of notification and mortality in PTB using age-standardized rates based on the World Standard Population. Notably, the results remained consistent when alternative age-standardized rates were utilized. Over the entire period from 2004 to 2021, the notification of PTB decreased from 46.41 per 100,000 to 28.53 per 100,000. We identified three joinpoints (2007, 2016, and 2019), resulting in four distinct periods with the following annual percentage changes:12.6% (95% CI: 4.8–21.0, *P* < 0.05) during 2004–2007, −7.3% (95% CI: −8.7 to −5.9, *P* < 0.05) during 2007–2016, 3.3% (95% CI: −11.2 to 20.0, *P* = 0.631) during 2016–2019, and −12.4% (95% CI: −26.4 to 4.2, *P* = 0.114) during 2019–2021 (Figure 1A; Table 3).

**Table 3:** Joinpoint incidence and mortality trend analysis of pulmonary tuberculosis.

**Type**	**Segment**	**Lower endpoint**	**Upper endpoint**	**APC**	**Lower CI**	**Upper CI**	**Test statistic (*t*)**	***P*-value**
Incidence	1	2004	2007	12.6^*^	4.8	21.0	3.9	< 0.05
	2	2007	2016	−7.3^*^	−8.7	−5.9	−11.9	< 0.05
	3	2016	2019	3.3	−11.2	20.0	0.5	0.631
	4	2019	2021	−12.4	−26.4	4.2	−1.8	0.114
Mortality	1	2004	2017	1.3	−3.6	6.3	0.6	0.588
	2	2017	2021	−20.5	−40.2	5.8	−1.7	0.106

^*^Indicates that the annual percent change (APC) is significantly different from zero at the alpha = 0.05 level. CI, confidence interval.

Regarding the age-standardized mortality rate (ASMRW) of PTB in Ningbo, it decreased from 0.07 to 0.06 per 100,000 during the period 2004–2021. We identified one joinpoint (2017), dividing the trend into two periods with the following annual percentage changes: 1.3% (95% CI: −3.6 to 6.3, *P* = 0.588) during 2004–2017, and −20.5% (95% CI: −40.2 to 5.8, *P* = 0.106) during 2017–2021 (Figure 1B; Table 3).

### 3.4 The impact of Ningbo 2011 PTB policy

**Figure 2:**
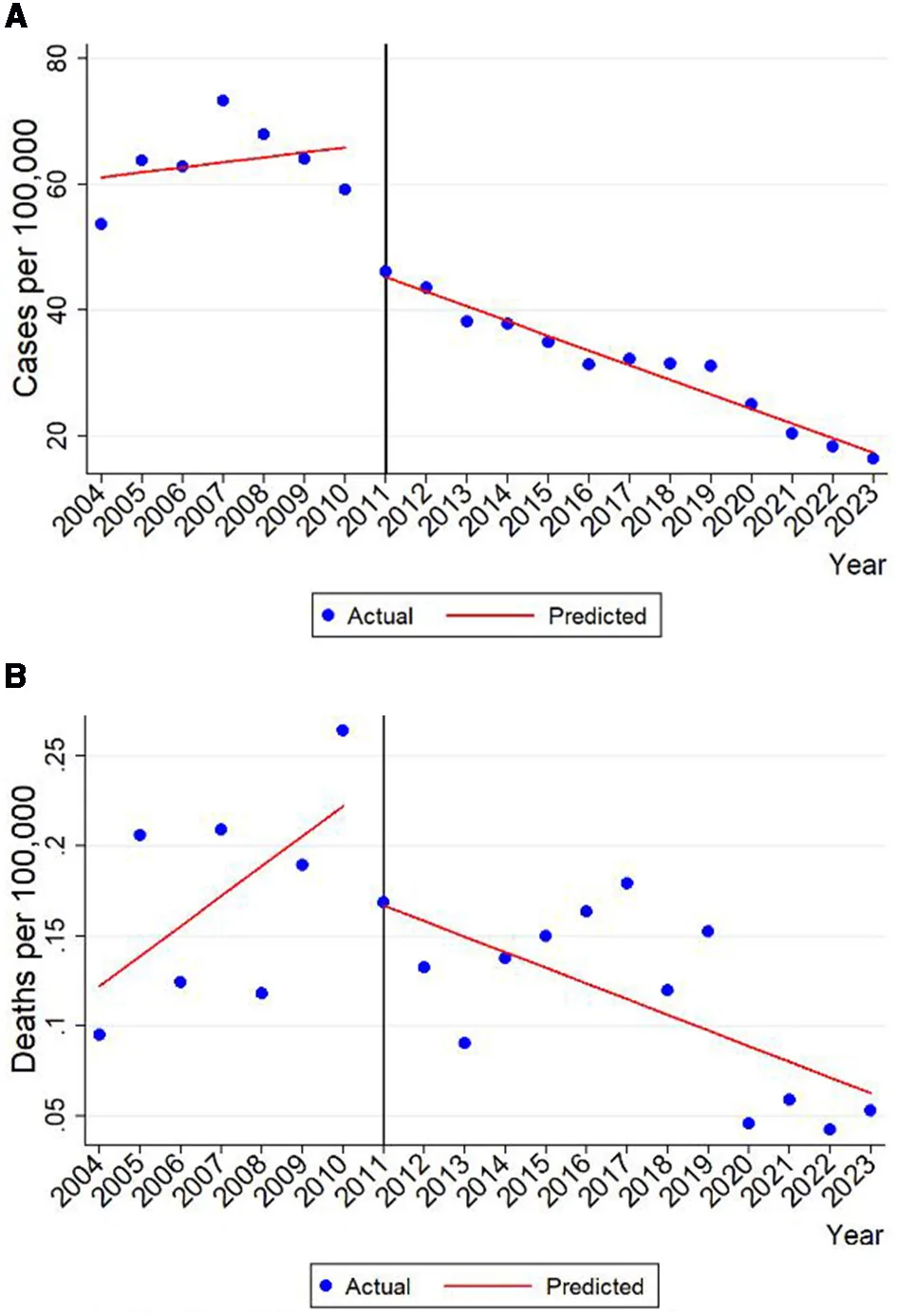
**(A, B)** An analysis of the impact of PTB policies in Ningbo City in 2011.

Figures 2A, B illustrate the estimated trend changes in PTB notification and mortality in Ningbo following the implementation of prevention policies in 2011. A notable reduction is observed in notification and mortality rates after introducing these policies. Our regression estimates indicate a decrease of 3.11 cases per 100,000 population in notification, representing a 7% reduction from the average of 43.33 cases. Mortality rates decreased by 0.025 deaths per 100,000 people, marking a 25% reduction from the average mortality rate of 0.10 deaths per 100,000 people. These results suggest that the 2011 prevention policies in Ningbo were highly effective in reducing PTB notification and mortality.

## 4 Discussion

Utilizing a web-based database sourced from the official Tuberculosis Management Information System, we gathered comprehensive data on PTB cases registered in Ningbo City, China, spanning from 2004 to 2021. From 2004 to 2021, China significantly enhanced its surveillance of PTB through the implementation of key policies and technological advancements. Notable initiatives included the establishment of an online reporting system in 2005, the introduction of a multidrug-resistant tuberculosis management plan in 2007, the initiation of targeted population screening in 2011, and the adoption of molecular diagnostic techniques in 2016. These enhancements improved both the sensitivity and specificity of detection methods, resulting in fluctuations in reported cases and revealing distinct epidemiological characteristics. Such developments have provided critical insights into the dynamics of tuberculosis transmission within the country. We analyzed these trends using age-standardized notification and mortality rates within an a joinpoint regression model. Notably, the PTB notification rate peaked at 65.48 per 100,000 in 2007 and thereafter witnessed a gradual decline at an annual average rate of 7.3% until 2016. Similarly, Zhejiang province exhibited a comparable pattern, with a peak notification of 89.5 per 100,000 in 2007, followed by a decline at an average annual rate of 4.8% from 2007 to 2015 (2). Our findings align with a recent study conducted in China between 2004 and 2019, which reported a decrease in the PTB notification rate from 84.67 per 100,000 in 2004 to 53.43 per 100,000 in 2019, with an average annual percentage change (APC) of −4.2%. Specifically, the notification rate slightly increased during 2004–2007 before declining steadily from 2007 to 2019, with the highest and lowest rates observed in 2005 (104.16 per 100,000) and 2019 (53.43 per 100,000), respectively (10).

It is pertinent to mention that the rising PTB notification in Ningbo during 2004–2007 may be attributed to previously underreported cases. Ningbo initiated the “Ningbo Tuberculosis Control Project” in 2002, aiming to enhance the detection rate of TB patients and ensure they receive regular treatment. In 2005, the Ningbo government further mandated all non-designated hospitals to implement a network direct reporting and referral system for TB patients and suspected cases, coupled with strengthened patient tracking mechanisms. Additionally, the city leveraged the world's largest internet-based TB management information system for reporting and managing confirmed TB cases (13). This internet-based reporting system significantly mitigated delays and incomplete reporting in communicable diseases, thereby facilitating effective PTB control. It enabled swift identification of PTB cases and ensured timely diagnosis and treatment for affected patients (14, 15). With these three initiatives in place, coupled with government funding, more PTB cases were detected and reported, potentially contributing to the temporary rise in PTB notification rates in Ningbo during 2004–2007.

Since 2007, the age-standardized notification rates of PTB have exhibited a gradual decline over time, potentially attributable to a series of government interventions and policies implemented. In 2007, Zhejiang Province successfully secured the Global Fund Tuberculosis Control project. The objective of this project is to enhance the management and treatment strategies for multidrug-resistant TB (MDR-TB) patients, aiming to effectively control the escalating number of MDR-TB cases (2). Since then, Ningbo has made significant advancements in optimizing, augmenting, and expanding screening procedures while also implementing measures to reduce or eliminate the financial burden associated with infectious disease treatment (16).

Between 2016 and 2019, the notification rate of PTB declined gradually in numerous countries (17, 18). During this period, Ningbo witnessed an average annual increase of 3.3% in age-standardized PTB notification rates, which can be attributed to the implementation of the China MOH-Gates Foundation TB Control Project. This project introduced advanced diagnostic technologies for PTB, including rapid molecular detection techniques like Xpert MTB/RIF, which improved the accuracy and reduced the detection time for *Mycobacterium TB* (M.tb) (19). Consequently, more patients with confirmed bacterial infections were identified and treated (2). Since the COVID-19 pandemic began, the TB notification rate has decreased by an average of 12.4% between 2019 and 2021, potentially due to preventive measures such as reducing gatherings and encouraging mask-wearing, which may have curbed TB transmission (20).

The PTB age-standardized mortality rate of Ningbo increased at an average rate of 1.3% during 2004–2017 and then decreased at an average rate of 20.5% during 2017–2021, the changes however were not statistically significant. In contrast, the age-standardized mortality China, as measured by PTB, exhibited a downward trend from 2005 to 2017, with an average annual percent change of −5.8%. This figure was peaked in 2005 (0.3 per 100,000) and was at its lowest in 2015 (0.15 per 100,000) (10). The inconsistent phenomenon may be because the number of TB deaths was rare in Ningbo and there is large randomness.

The highest crude notification rate was reported for the age group of 20–29 years followed by the age group of 70–79 years (>80 per 100,000). Adolescence is characterized by an elevated susceptibility to TB. Although the precise mechanisms remain incompletely elucidated, it is postulated that sex hormones, alterations in social contact patterns, and immunological changes may each have exerted a contributory influence (21). The crude mortality rate increases with age, and people over 75 years of age are vulnerable. Older individuals with pulmonary TB, characterized by compromised immune function and a higher prevalence of chronic comorbidities, exhibit an increased notification of atypical manifestations, heightened susceptibility to adverse drug reactions, and elevated mortality rates associated with TB compared to their younger counterparts (22–24). The aging process is expected to continue exerting a significant impact on the escalation of PTB-related mortality (25, 26). The data indicates that elderly patients with PTB exhibit a higher mortality rate compared to those without PTB. However, if promptly diagnosed and appropriately treated, these elderly patients with PTB do not exhibit higher mortality rates compared to those without the condition (27).

The advantages of our study are as follows: (1) this is the first study to investigate the age-standardized notification and mortality rates of PTB in Ningbo. (2) This study covered all the newly diagnosed PTB and death cases in long term, which could represent the epidemiological trend of PTB in Ningbo. (3) Our findings provided direct evidence of PTB control and prevention for Ningbo Municipal Center for Disease Control and Prevention. However, our study had some limitations. First, our data came from the yearbook, and more detailed demographic information of each patient was not available, meaning we cannot examine the association of notification rate with these characteristics. Second, only intervention and policy factors affecting notification and mortality were analyzed, and environmental and social factors were not analyzed. Third, this study used PTB notification rates rather than total TB incidence rates because China's infectious disease reporting system does not mandate EPTB reporting. This approach may underestimate the overall TB burden, particularly among children who have a higher proportion of EPTB cases, potentially affecting policy evaluation for specific populations. Additionally, we did not differentiate between drug-sensitive and drug-resistant TB cases, which limits our assessment of targeted interventions like the 2007 MDR-TB control project. Future studies should incorporate drug resistance data to enhance policy evaluations.

## 5 Conclusion

Since 2004, the notification and mortality rates of PTB in Ningbo have witnessed a decline due to the effective implementation of various measures and projects. However, there still exists a substantial disparity when compared to the 2035 targets outlined in the End TB Strategy. Reinforcement of existing measures and the development of novel strategies for prevention, diagnosis, and treatment are imperative to further reduce the notification of PTB. Moreover, males and elderly individuals appear to be more vulnerable to both the notification and mortality of PTB, highlighting the need for targeted implementation of future control measures within these specific population cohorts.

## Data Availability

The original contributions presented in the study are included in the article/supplementary material, further inquiries can be directed to the corresponding author.

## Footnotes

^1^ http://www.surveillance.cancer.gov/joinpoint/

## Appendix

**Characteristics of patients with TBP**

Table A1 presents the baseline characteristics of pulmonary tuberculosis (PTB) cases and deaths from 2004 to 2021. A total of 68,392 PTB notification cases were recorded, with 68.12% being males. During the same period, there were 236 deaths, 81.36% of which were males. The mortality rate among PTB cases was 0.35% (236/68,392). The majority of PTB cases were observed in adults aged 15–34 (~42%) and 35–64 (~43%). A minimal number of cases (0.49%) were aged 14 or younger, while ~15% were aged 65 or above.

Regarding deaths, more than half occurred in individuals aged 65 or above. The remaining deaths were distributed among those aged 35–64 (33.47%), 15–34 (8.90%), and below 15 (1.69%). In summary, the demographic information highlights the predominance of PTB cases and deaths among older adults, with a slight majority being male.

**Table A1:** Baseline characteristics of the pulmonary tuberculosis cases and deaths.

**Variables**	**Group**	**Incidence cases**	**Death cases**
	**Total**	**68,392**	**236**
Gender	Male	46,592 (68.12%)	192 (81.36%)
	Female	21,800 (31.88%)	44 (18.64%)
Age	Aged ≤ 14	337 (0.49%)	4 (1.69%)
	15–34	28,967 (42.35%)	21 (8.90%)
	35–64	29,097 (42.54%)	79 (33.47%)
	≥65	9,991 (14.61%)	132 (55.93%)
